# Supplementary figures and images for: A simple assay for inhibitors of mycobacterial oxidative phosphorylation
Source: J Biol Chem. 2023 Nov 20;300(1):105483. doi: 10.1016/j.jbc.2023.105483 (PMC10770618; doi:10.1016/j.jbc.2023.105483)

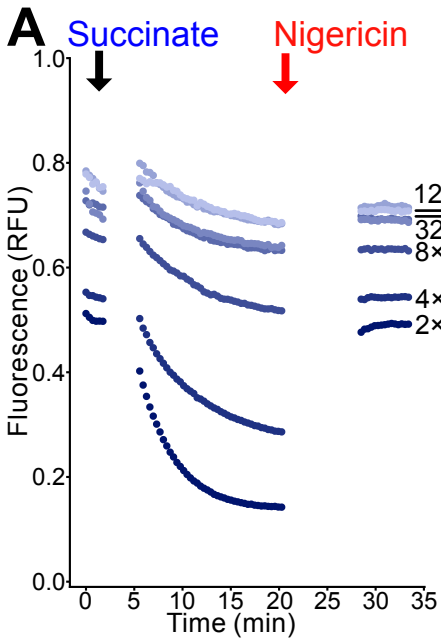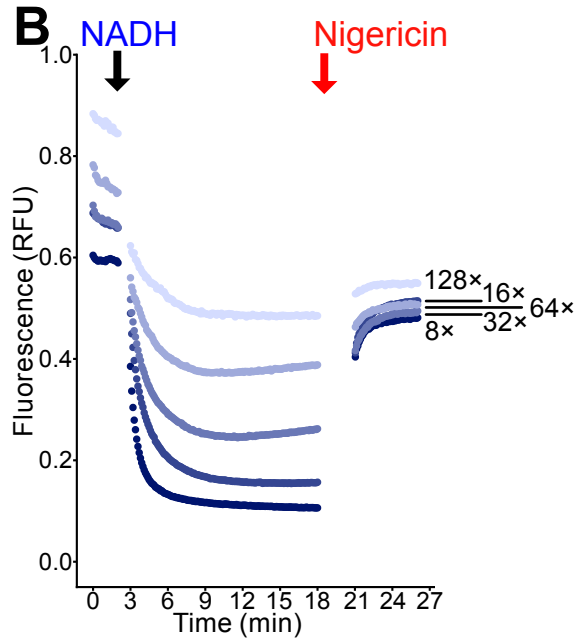

Supplement: Supplementary Figure 1 — Raw fluorescence traces for dilution of IMVs.A, succinate-driven IMV acidification. B, NADH-driven acidification. [file mmc1.pdf]

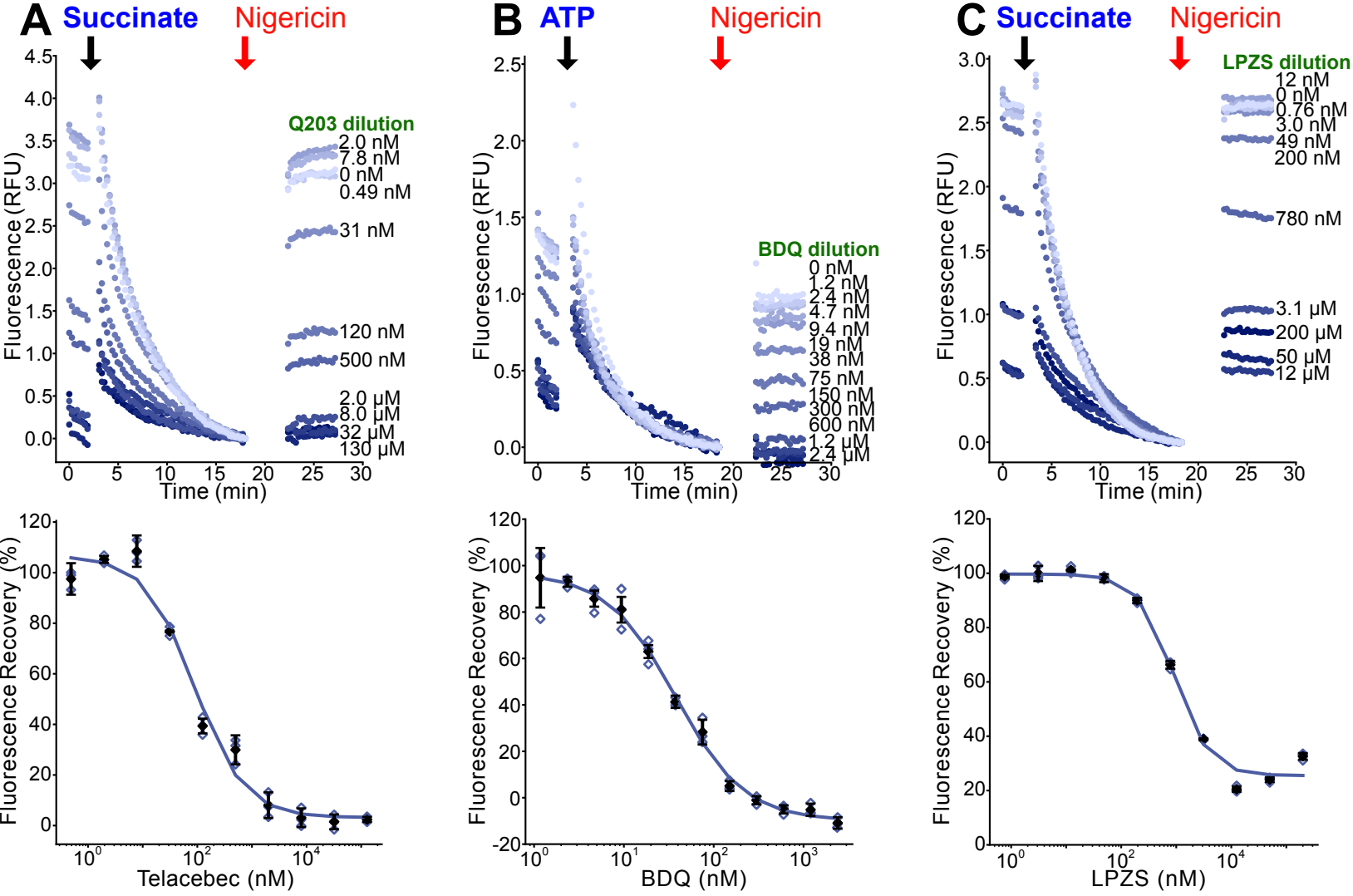

Supplement: Supplementary Figure 2 — Replication of inhibitor dilution experiments.A, replicate experiment for telacebec (Q203) inhibition of succinate-driven IMV acidification using an independently prepared dilution series of telacebec. The experiments indicate an IC50 of ~90 nM. B, replicate experiments for bedaquiline (BDQ) inhibition of ATP-driven IMV acidification using an independently prepared dilution series of bedaquiline. The experiments indicate an IC50 of ~40 nM. C, replicate experiments for lansoprazole sulfide (LPZS) inhibition of succinate-driven IMV acidification using an independently prepared dilution series of LPZS. The experiments indicate an IC50 of ~890 nM. Open symbols show technical replicates. Filled symbols show the mean from n = 3 technical replicates. Error bars indicate ±SD when shown. [file mmc2.pdf]
